# Topology-Preserving Circular Emotion Representation for Modern Dance via Interpretable Laban Prototypes

**DOI:** 10.3390/biomimetics11100755

**Published:** 2026-10-19

**Authors:** Kai Qiu, Zhihao Gong, Zhidong Wang, Fan Yang, Lei Fang

**Affiliations:** 1 Department of Dance, Graduate School, Sangmyung University, Seoul 03016, Republic of Korea; 202336008@sangmyung.kr (K.Q.); 120162736@wut.edu.cn (Z.G.); 202434038@sangmyung.kr (Z.W.); 2 School of Film and Television Media, Wuchang University of Technology, Wuhan 430223, China; 3 Department of Emotion Engineering, Sangmyung University, Seoul 03016, Republic of Korea; 202432076@sangmyung.kr

**Keywords:** Laban Movement Analysis (LMA), Russell circumplex model (RCM), circular emotion representation, modern dance, kinematic descriptors, continuous emotion representation

## Abstract

Modern dance conveys complex emotions through continuous bodily movement, but assigning recorded motion to continuous affective coordinates remains challenging. This study examines the mapping of Laban Movement Analysis (LMA)-informed kinematic descriptors to predefined emotion prototypes in Russell’s valence–arousal space. Thirty-one frame-level descriptors were extracted from recorded dance motion and temporally aggregated for circular-target regression. Four-quadrant classification and direct valence–arousal regression were also evaluated as separate modeling tasks. In the 12-category circular experiment, the selected ExtraTrees model was evaluated on 117 motion sequences using five-fold stratified cross-validation and obtained an angular mean absolute error of 36.765 ± 6.414° and a quadrant macro-F1 of 0.670 ± 0.094. These values measure prediction against assigned category-level affective targets. Shortest-arc interpolation of predefined prototype angles, combined with linear interpolation of their radii, produced continuous numerical paths in valence–arousal space; generated skeletal transitions were not evaluated. A separate questionnaire involving 36 observers assessed 24 videos of the recorded dancers. The findings provide preliminary evidence that the selected descriptors recover assigned affective direction in this recorded-dance dataset. Perceptual evaluation of generated skeletal or avatar motion and investigation of potential applications in immersive or digital-human settings remain future work.

## 1. Introduction

### 1.1. Background and Motivation

Modern dance conveys affect through evolving bodily movement, including changes in posture, movement dynamics, and spatial organization. Describing these qualities computationally requires motion features that remain interpretable while supporting the analysis of relationships among emotional states. Russell’s circumplex model of affect represents emotion in a two-dimensional valence–arousal space and provides a theoretical basis for examining both the position and the directional relationships of affective states [1,2,3].

Human movement can convey expressive information through the coordination of body parts, the dynamic qualities of action, changes in body form, and the organization of movement in space. Laban Movement Analysis (LMA) describes these observable aspects through four related components: Body, Effort, Shape, and Space [4,5]. Body concerns the participation and coordination of body segments; Effort concerns movement dynamics and is described through the factors of Time, Weight, Flow, and Space; Shape concerns changes in body form, such as expansion and contraction; and the broader Space component concerns spatial pathways, levels, and the mover’s use of surrounding space. Space as an Effort factor describes the directness of directional attention and should therefore be distinguished from Space as a broader LMA component. In the present study, these concepts guide the construction of kinematic descriptors from recorded skeletal motion. The 31 implemented descriptors are analyzed in three feature groups: Effort (four dimensions), Shape (four dimensions), and combined Body/Space (23 dimensions). This computational translation of observable human movement provides the basis for the proposed biomimetic representation; the descriptors are not treated as direct measurements of underlying biological mechanisms.

Previous studies have used LMA-inspired descriptors to analyze emotional similarity and classify dance movements [6], to estimate continuous affective trajectories from expressive body movement [7], and to relate dance-motion features to emotion coordinates for motion stylization [8]. These contributions establish the relevance of LMA and the valence–arousal framework to affective movement analysis. The question addressed in the present study is more specific: how effectively can interpretable modern-dance motion descriptors recover the directional organization of RCM affective space, and for which emotion categories does that mapping remain uncertain?

This question requires attention to both representation and evaluation. Predicting valence and arousal independently provides continuous coordinates but does not explicitly encode the periodic relationship between angular positions near 0° and 360°. An angular–radial representation makes this relationship explicit and permits circular angular error to be assessed alongside conventional coordinate-prediction measures. Moreover, because dance movements unfold over time, the effects of LMA feature composition and temporal aggregation must be examined when interpreting the resulting affective mapping.

### 1.2. Research Objectives and Evaluation Scope

Building on earlier LMA-based affect analysis and continuous movement-to-emotion mapping, the framework examines whether explicit angular and radial targets can represent the directional structure of affective space from recorded dance motion. Its relationship to existing approaches is discussed in Section 2.1. The aim is to characterize both the affective structure recoverable from the motion features and the categories for which prediction remains less reliable. To this end, the study analyzes a 31-dimensional set of LMA-derived motion descriptors, grouped as Effort, Shape, and Body/Space features in the implemented experiments. Their relationship to RCM space is evaluated through four-quadrant classification, direct valence–arousal regression, and circular-target regression. Component-level ablation and temporal-aggregation analyses are used to examine how feature composition and observation scale affect the results. For circular-target prediction, affective direction is represented by cosθ and sinθ, while *r* represents affective intensity. The resulting predictions are assessed using coordinate-level and angular-error measures, including category-level deviations from predefined emotion prototypes.

Assigned prototypes are also interpolated in polar form. The resulting curve is a property of the predefined (θ,r) targets; it is neither a pose generator nor evidence that an affective crossing would look coherent. The questionnaire, described in Section 3.5, uses the original performance videos and is kept separate from the kinematic model.

### 1.3. Main Contributions and Organization

The main contributions of this paper are threefold.

- An explicit circular-target representation for modern-dance affect analysis: The study represents affective direction using cosθ and sinθ and models intensity through *r*. It evaluates the resulting circular-space mapping alongside four-quadrant classification and direct valence–arousal prediction, with particular attention to directional and category-level errors.
- An interpretable analysis of LMA-derived motion features: The study examines 31 motion descriptors through component-level ablation and temporal-aggregation experiments. These analyses identify how the implemented Effort, Shape, and Body/Space feature groups and different observation scales relate to affective prediction in the present dataset.
- Polar interpolation of predefined prototypes, reported only as coordinate trajectories, together with a separate observer study of the original performance videos. The observer study checks label plausibility. It does not validate the descriptors in isolation or any generated motion.

The remainder of this paper is organized as follows. Section 2 reviews affective movement research, discusses the requirements of modern-dance emotion modeling, and positions the present study relative to existing approaches. Section 3 describes the RCM–LMA circular-space modeling framework. Section 4 reports the feature analyses, classification and regression results, circular-space evaluation, prototype interpolation, and perceptual questionnaire. Section 5 discusses the findings and limitations, and Section 6 concludes the paper.

## 2. Related Work

### 2.1. Emotion Understanding in Dance Scenarios

Emotion understanding constitutes a central concern in psychology, computer animation, and human–computer interaction [2,9]. At its core lies the challenge of converting subjective affective states into quantifiable representations of bodily movement. Russell’s circumplex model [1] situates emotion within a continuous two-dimensional space of valence and arousal, thereby offering a theoretical basis for visualizing dynamic affective trajectories in dance and theatrical performance. Complementing this geometric framework, Laban Movement Analysis (LMA) organizes the observation of movement through the broader components of Body, Effort, Shape, and Space. Within the Effort component, Space, Weight, Time, and Flow are four factors describing qualities of action; they are not the four components of LMA [10,11].

Early studies predominantly addressed discrete emotion classification. Aristidou and colleagues [8,12,13] employed LMA features to examine the affective similarity of dance movements within Russell’s dimensional space. Although high accuracy was achieved on controlled datasets, generalization remained constrained by inter-individual variability and spatial overlap, and the approach struggled to capture affective continuity. Neural systems inspired by LMA, as explored by Yiannakides et al. [14], Carreno-Medrano et al. [15], and Pavllo et al. [16], can generate continuous emotion curves in Russell space in real time; yet, performance degrades markedly under noise or cross-cultural conditions, and high-dimensional uncertainty is insufficiently modeled. Shiratori et al. [17,18] synthesized music-matched dance sequences on the basis of Laban theory, while Nicolaou et al. [19] predicted valence and arousal from facial expression, shoulder motion, and audio cues.

These lines of work, however, relied on overly coarse simplifications in both motion simulation and symbolic representation, largely overlooking the accumulated insights of dance semiotics. More recent efforts have emphasized robustness. The LSTM architectures proposed by Wei [20] and Charalambous [21] integrate multi-level LMA features and incorporate Bayesian inference to account for uncertainty. A meta-analysis by Andreou et al. [22] identified correlations between parameters such as spinal inclination and joint energy with affective intensity, yet stopped short of providing a complete, dance-specific real-time computational pipeline, and parameter extraction remained inefficient. Other researchers have employed subsets of LMA components to interpret bodily expressions of humanoid robots [23,24,25]; nevertheless, modeling capacity and computational efficiency for multi-actor interaction continue to present limitations.

Prior work has made dance affect measurable, and some of it already emits continuous Russell-space curves under a real-time constraint. That deployment question is not taken up here. The present experiments ask whether the 31 kinematic descriptors recover the directional layout of predefined prototypes, and for which of the 12 categories the recovery fails.

### 2.2. Characteristics and Requirements of Emotional Expression in Modern Dance

Modern dance, as a central medium of contemporary performing arts, relies primarily on whole-body dynamic movement to convey emotion. Unlike the codified vocabulary of traditional dance forms, it prioritizes fluid, improvisational, and highly individualized body trajectories that directly externalize inner psychological states. This mode of expression depends far more on the qualitative texture of movement than on fixed postures or facial detail. Within LMA, Effort, Shape, Space, and Body give a vocabulary for observable movement qualities. Prior work has used that vocabulary to relate dance kinematics to valence–arousal coordinates and to spectators’ discrete emotion judgments [7,10,26,27,28,29].

To achieve a satisfactory simulation of complex human body language, a movement description that is as simple as possible yet sufficiently rich is required; LMA satisfies these dual constraints. The application of LMA concepts to the quantification of emotional content has become increasingly widespread in motion analysis and retrieval [30,31,32,33]. A shared objective across these applications is the extraction of movement style. Early studies employed the LMA framework to quantify emotion in contemporary dance performances. Herath et al. [34] and Aristidou et al. [12] extracted LMA entities from controlled contemporary-dance data and attained high accuracy in discrete emotion classification under the Russell circumplex model; their methods, however, remained largely restricted to segmented excerpts, limiting their ability to capture the continuous dynamic evolution of complete performances and constraining generalization across dancer-specific styles and improvisational variation. Senecal et al. [7] proposed a continuous body-emotion recognition framework for theatrical performance that maps LMA features onto Russell’s two-dimensional space via neural networks and visualizes evolving emotional trajectories. Although this work advanced dynamic modeling, it depends heavily on annotated data and exhibits markedly reduced robustness under real-stage conditions involving noise, occlusion, or multi-performer interaction.

## 3. RCM–LMA Circular-Space Modeling Framework

**Figure 1:**
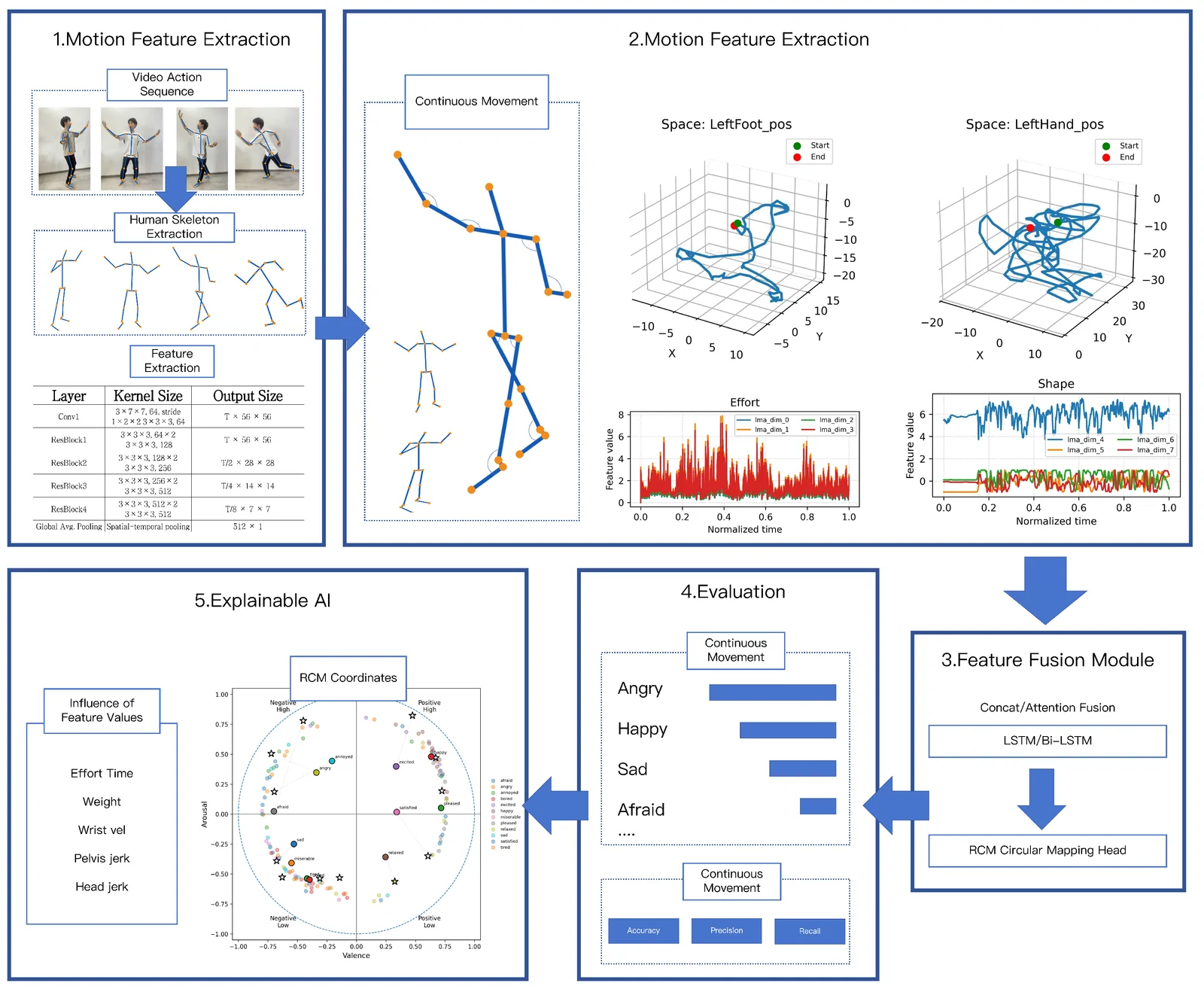
Overall framework. Solid blue arrows indicate the workflow, while dotted blue outlines group related elements. Orange circles and blue lines denote skeletal joints and connections; green and red markers indicate trajectory start and end points. Plot colors correspond to their legends. In the RCM plot, small points represent sequence predictions, large labeled points represent category means, and stars indicate assigned prototypes. The ellipsis denotes the remaining eight emotion categories.

Recorded dance sequences are mapped to predefined prototypes in Russell’s valence–arousal space. Continuity is then inspected by interpolating those assigned prototypes in polar coordinates. The interpolant is a coordinate-space construction, not a transition between generated poses. Figure 1 shows the five offline stages used in the experiments: preprocessing, kinematic representation, LMA-derived descriptors, prototype mapping, and polar interpolation of the assigned (θ,r) targets.

### 3.1. Construction of Kinematic Representation

Modern dance constitutes a highly affective form of non-verbal expression, in which movement sequences convey complex psychological experiences through continuous bodily dynamic narratives. The computational representation used here starts from the recorded global three-dimensional joint positions of each BVH sequence.

Let $p_{q,j}\left(t\right)$ denote the global position of joint *j* at frame *t* of sequence *q*, and let Δt denote the frame interval read from the corresponding BVH file. Velocity and acceleration are computed by successive finite differences:

(1) $$v_{q,j}\left(t\right)=\frac{p_{q,j}\left(t\right)-p_{q,j}\left(t-1\right)}{\Delta t},\;\;\;\;\;\;a_{q,j}\left(t\right)=\frac{v_{q,j}\left(t\right)-v_{q,j}\left(t-1\right)}{\Delta t}.$$

The first-frame velocity and acceleration are set to zero. The extracted frame-level descriptors are then grouped by their source motion file to construct the complete-sequence inputs described above.

### 3.2. Quantification of LMA-Derived Kinematic Features

For sequence *q*, the 31 frame-level descriptors are computed from $p_{q,j}\left(t\right)$ and $a_{q,j}\left(t\right)$, with joints indexed by alphabetically sorted names. The descriptors are grouped as Effort (dimensions 0–3), Shape (4–7), and Body/Space (8–30). The Effort-group dimensions are weighted versions of one acceleration statistic:

(2) $$f_{q,k}\left(t\right)=w_{k}\frac{\frac{1}{J}\sum_{j=0}^{J-1}∥a_{q,j}\left(t\right)∥}{{\mathrm{std}}_{j}\;\left(∥a_{q,j}\left(t\right)∥\right)+10^{-8}},\;\;\;\;\;\;\left(w_{0},w_{1},w_{2},w_{3}\right)=\left(0.8,1.2,0.9,1.1\right).$$

Thus, dimensions 0–3 are acceleration-based proxies, not separate measurements of the four Effort factors. Shape dimensions 4–7 contain the logarithm of the selected joints’ bounding-box volume and the three components of a normalized vector between the first and last selected joints. Joint indices 5–11 are selected when the skeleton has more than 12 joints; otherwise, the available joints up to index 11 are used. Body/Space dimensions 8–17 contain ten flattened coordinates relative to joint 0, and dimensions 18–30 contain thirteen pairwise distances among joints 0–5 in index order. Body and Space are not computed or evaluated as separate feature groups.

Two additional columns are saved: lma_mean, the frame-level arithmetic mean of the 31 descriptors, and intensity, obtained by dividing lma_mean by its maximum over the merged frame-level dataset. Neither column represents the prototype radius $r_{e}$.

For each complete sequence, 15 temporal statistics are computed for each of these 33 columns: mean, standard deviation, median, minimum, maximum, range, 25th and 75th percentiles, interquartile range, 10th and 90th percentiles, mean absolute consecutive-frame difference, standard deviation of consecutive-frame differences, mean squared value, and linear trend. This yields a 33×15=495-dimensional input $x_{q}$. Missing-value imputation and standardization are fitted within each cross-validation training fold. The selected ExtraTrees model uses all 495 input dimensions to learn $x_{q}\mapsto y_{q}$ from the assigned category targets. The mapping to valence–arousal coordinates is therefore learned by regression rather than specified by a direct formula for each LMA component.

### 3.3. RCM Four-Quadrant Label Construction

#### 3.3.1. Emotion Prototype Vector Mapping

Each dance sequence *q* is associated with the source emotion label $e_{q}$ of its recorded motion file. For the enhanced circular-mapping experiment, each of the 12 included emotion categories is assigned a predefined prototype angle $\theta_{e}$ and radius $r_{e}$, as listed in Table 1. These values specify the supervised affective target; they are not estimated from frame-wise valence–arousal annotations or from the extracted motion features. Angles listed in degrees are converted to radians for the following calculations. The Cartesian coordinates and circular regression target for sequence *q* are

(3) $$\left(V_{q},A_{q}\right)=\left(r_{e_{q}}\cos\theta_{e_{q}},\;r_{e_{q}}\sin\theta_{e_{q}}\right),\;\;\;\;\;\;y_{q}=\left(\cos\theta_{e_{q}},\;\sin\theta_{e_{q}},\;r_{e_{q}}\right).$$

The model predicts three values $\left({\hat{c}}_{q},{\hat{s}}_{q},{\hat{r}}_{q}\right)$. Its two-dimensional valence–arousal prediction is obtained by normalizing the predicted direction and constraining the radius to the interval [0, 1]:

(4) $$d_{q}=\max\;\left(\sqrt{{\hat{c}}_{q}^{\;2}+{\hat{s}}_{q}^{\;2}},\;10^{-8}\right),\;\;\;\;\;\;\;\left({\hat{V}}_{q},{\hat{A}}_{q}\right)=\frac{\mathrm{clip}({\hat{r}}_{q},0,1)}{d_{q}}\left({\hat{c}}_{q},{\hat{s}}_{q}\right).$$

The predicted angle is ${\hat{\theta }}_{q}=\mathrm{mod}\;\left(\mathrm{atan2}({\hat{A}}_{q},{\hat{V}}_{q}),2\pi \right)$. Representing angular direction by its cosine and sine makes the targets at 0° and 360° equivalent. The prototype radius $r_{e}$ denotes the assigned affective intensity of an emotion category; it is distinct from the frame-level input column named intensity, which is defined below.

#### 3.3.2. Circular Continuous Interpolation Mechanism

Assigned prototypes are connected in polar form to inspect coordinate-space continuity. Let $\left(\theta_{s},r_{s}\right)$ and $\left(\theta_{t},r_{t}\right)$ be the source and target angle–radius pairs. For u∈[0, 1], the path uses the shortest signed angular difference and a linear radius:

(5) $$\Delta \theta =\mathrm{mod}\left(\theta_{t}-\theta_{s}+\pi ,2\pi \right)-\pi ,\;\;\;\;\;\;\theta \left(u\right)=\theta_{s}+u\Delta \theta ,\;\;\;\;\;\;r\left(u\right)=r_{s}+u\left(r_{t}-r_{s}\right).$$

(6) $$\left(V(u),A(u)\right)=\left(r(u)\cos\theta (u),\;r(u)\sin\theta (u)\right).$$

For each source–target pair, the path (V(u),A(u)) was evaluated at K=80 uniformly spaced values $u_{j}=j/\left(K-1\right)$, j=0,…,K−1. The prototype angles and radii determine the continuous parametric curve, whereas *K* sets the resolution of its sampled representation. Adjacent-sample angular changes and coordinate second differences were calculated from the sampled valence–arousal coordinates. The resulting statistics describe the numerical continuity of the assigned affective-coordinate paths. No intermediate descriptor, skeletal pose, or avatar frame is produced. Each path is a closed-form sample of Equations (5) and (6) at K = 80, and the regressor is applied once to the 495-dimensional sequence aggregate. Both steps were run offline. The experiment contains no render loop, so rendering latency, inference budget, and frame rate are not defined for this study and are not reported.

### 3.4. Evaluation Metrics for Circular Predictions and Prototype Paths

For a target angle $\theta_{q}$ and a predicted angle ${\hat{\theta }}_{q}$, both expressed in radians, sequence-level angular error is calculated using wrapped circular distance and reported in degrees:

(7) $$d_{\mathrm{circ}}\left({\hat{\theta }}_{q},\theta_{q}\right)=\frac{180}{\pi }\left|\mathrm{atan2}\;\left(\sin\left({\hat{\theta }}_{q}-\theta_{q}\right),\cos\left({\hat{\theta }}_{q}-\theta_{q}\right)\right)\right|.$$

Angular MAE is the mean of this error over the held-out sequences in each cross-validation fold. The final result is reported as the mean and sample standard deviation of the five fold-level MAEs.

Category-level angular deviation is calculated separately. For emotion category *e*, the predicted category angle ${\bar{\theta }}_{e}$ is the circular mean of the predicted sequence angles, and its angular deviation is measured against the assigned prototype angle $\theta_{e}$:

(8) $${\bar{\theta }}_{e}=\mathrm{mod}\;\left(\mathrm{atan2}\left(\frac{1}{N_{e}}\sum_{q:e_{q}=e}\sin{\hat{\theta }}_{q},\;\frac{1}{N_{e}}\sum_{q:e_{q}=e}\cos{\hat{\theta }}_{q}\right),2\pi \right),\;\;\;\;\;\;D_{e}=d_{\mathrm{circ}}\left({\bar{\theta }}_{e},\theta_{e}\right).$$

The category-level angle $D_{e}$ is not the mean of the individual sequence errors. The Cartesian centroid error is a different quantity: the Euclidean distance between the mean predicted valence–arousal coordinates of category *e* and its assigned prototype coordinate.

For an interpolated prototype path sampled at K=80 points, max jump is the largest absolute angular change between adjacent sampled points, reported in degrees. Smoothness is the mean Euclidean norm of the second differences of the sampled valence–arousal coordinates. Both describe the numerical coordinate path at the stated sampling resolution, rather than the perceptual smoothness of generated skeletal motion.

### 3.5. Subjective Perception Validation Questionnaire

A supplementary questionnaire was used to examine whether the assigned emotion labels remained intelligible to observers of the recorded performances. It was not used for training, feature selection, or parameter tuning. Stimuli were 2D RGB clips of the original dancers, not untextured avatars, skeleton renderings, or VR views. Ratings may therefore reflect facial expression, body appearance, clothing, lighting, and scene context in addition to whole-body kinematics. The questionnaire is an external check on label plausibility and observer agreement. It is not a perceptual validation of the 31 LMA descriptors, of generated transitions, or of digital-human display.

As summarized in Table 2, a total of 36 participants were recruited (age range 18–41 years, mean 26.75±5.72 years), comprising 17 females (47.2%), 17 males (47.2%), and 2 participants who preferred not to disclose gender (5.6%). Professional background and dance experience were also recorded to characterize the sample composition and to aid interpretation of rating discrepancies.

The stimulus set comprised 24 RGB clips of the original dancers, 2 clips per target emotion, presented in random order. Each clip was taken from a longer recording and truncated to 12–16.5 s so that it contained one contiguous movement phrase while remaining short enough for 36 observers to rate 24 items on four scales without excessive fatigue. This window is comparable to a single dance phrase rather than to a full narrative arc. No skeleton-only or neutral-avatar condition was included; ratings may therefore reflect facial appearance, body shape, clothing, lighting, and scene context as well as whole-body kinematics.

Four ratings were collected for each video: valence, arousal, expressive clarity, and the closest emotion category. The first three dimensions were assessed on seven-point Likert scales (valence: very negative–very positive; arousal: very calm–very activated; clarity: very unclear–very clear). Category selection permitted an “unclear” option to avoid forced classification when expression was ambiguous.

Data analysis encompassed reliability assessment, rating-difference testing, category-consistency analysis, and model–subjective consistency evaluation. Cronbach’s α was computed to evaluate inter-rater reliability for valence, arousal, and clarity. Separate one-way repeated-measures ANOVAs were conducted with each of the three continuous ratings as the dependent variable and the emotion category as the within-subject factor. Sphericity was examined with Mauchly’s test; when the assumption was violated, Greenhouse–Geisser corrected results were reported. Post hoc pairwise comparisons employed Bonferroni-corrected paired *t*-tests, with exploratory multiple comparisons further controlled by the Benjamini–Hochberg FDR procedure.

Theoretical prototype coordinates, model predictions, and mean observer ratings were compared only at the category level. Directional agreement was taken as evidence that a label was perceptually stable for these clips, not that the LMA feature set caused the rating. Moderate offsets were treated as boundary cases; large offsets were left uninterpreted as prototype support. Observer means were not treated as ground truth for the kinematic model.

## 4. Results

### 4.1. Dataset Statistics

#### 4.1.1. Dataset Statistics Results

Table 3 summarizes the Custom Acted Dance Motion Capture Dataset. Folder-level counts are unbalanced. The singleton folders active and curious (one sequence each) are listed only for completeness and are excluded from four-quadrant classification, valence–arousal regression, circular-target regression, and category-level error analysis. The mixed-label folder (mix, five sequences) is likewise excluded from supervised mapping. The remaining 12 categories yield 117 sequences, with 8–12 sequences per class. All reported scores are therefore estimates on this restricted, still small corpus; they are not estimates for the discarded folders or for a balanced 12-class population.

Sequence-length statistics reveal substantial variation in mean frame counts across emotion categories, ranging from 5137 to 12,641 frames. Moreover, the standard deviation exceeds 4000 frames for most categories, indicating considerable intra-class variability in temporal structure. This heterogeneity demonstrates that the dataset preserves the diversity of authentic dance movements while exhibiting marked differences in sequence duration.

**Figure 2:**
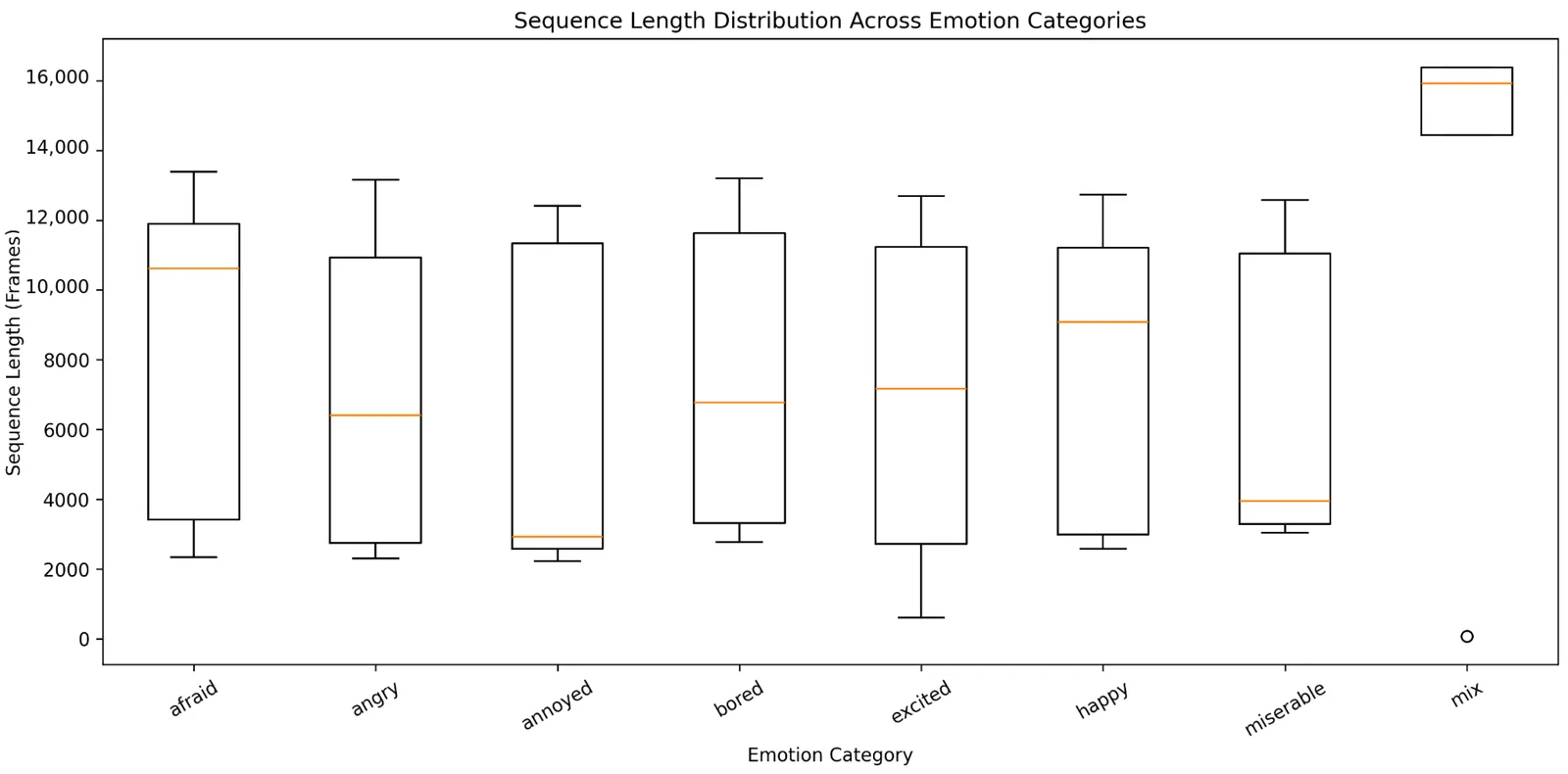
Sequence length distribution across emotion categories in the Custom Acted Dance Motion Capture Dataset. Categories with fewer than three samples (i.e., active and curious) were excluded from the boxplot because reliable distributional statistics could not be derived from single observations. The boxplots show the median, interquartile range, and outlier samples for each remaining emotion category.

Figure 2 presents the sequence-length distributions of emotion categories with sufficient sample sizes. Clear inter-category differences are evident, accompanied by pronounced intra-category dispersion in the majority of classes. Categories such as afraid, angry, bored, and happy display wide length ranges, reflecting substantial diversity in movement organization under the same affective condition, whereas annoyed and miserable exhibit more concentrated distributions, suggesting greater consistency in action duration.

#### 4.1.2. Datasets and Preprocessing

This study uses a custom acted dance motion-capture set [6,8,29]. Several dancers performed under prescribed emotion instructions; motion was stored as BVH, with synchronized MP4 clips kept for verification. Folder names supply the sequence labels. The protocol therefore elicits category-typical movement rather than unrehearsed stage improvisation. Shared cues across dancers—expansion for positive-high items, collapse for negative-low items—may inflate within-corpus separability relative to spontaneous modern dance, where the same label can be carried by smaller kinematic differences. Subject identifiers were not used as a blocking factor; the five-fold splits in Section 4 are stratified by emotion folder, not by dancer or phrase vocabulary.

Data preprocessing began with integrity checks on all BVH files, discarding samples that exhibited corrupted skeletal structures, missing frame data, or parsing failures. Temporal intervals were then standardized according to the frame-time information, and local joint rotations were converted into global joint positions via forward kinematics. To eliminate the influence of body-size differences, a root-node normalization strategy was applied: joint positions were transformed into a local coordinate system relative to the root node and subsequently scaled by torso length. Anomalous frames were detected on the basis of velocity and acceleration magnitudes and suppressed through local smoothing to mitigate jitter and discontinuities. After preprocessing, all samples were converted into standardized kinematic sequences that served as the input for subsequent feature extraction and emotion modeling.

### 4.2. Model Robustness Validation Experiments

#### 4.2.1. Statistical Analysis of LMA Features

Sequence-level LMA representations reorganized by RCM four-quadrant labels further confirm the discriminative power of the proposed features within the structure of the affective space. Table 4 reports the LMA features that exhibit the most pronounced differences across RCM quadrants. Multiple features retain significant between-group differences after FDR correction, with the significant ones concentrated primarily in the Shape and Body/Space components. In particular, Shape features such as lma_dim_4 and lma_dim_6 display relatively high test statistics and effect sizes, indicating that body expansion, volumetric change, and directional tendency play important roles in distinguishing affective quadrants. Several Body/Space features likewise show significant differences, suggesting that relative joint configurations and spatial organization patterns referenced to the root node supply effective movement-semantic information for the structure of the affective space.

**Figure 3:**
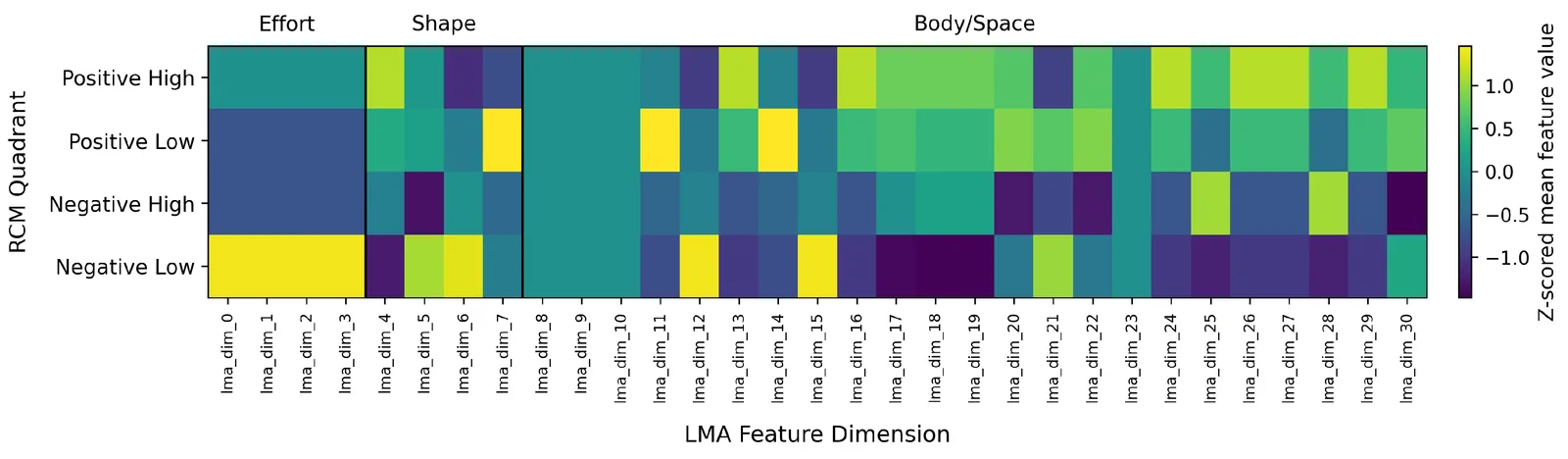
Top LMA features showing significant differences across RCM quadrants.

Figure 3 presents the standardized mean patterns of the 31-dimensional LMA features across the four RCM emotion quadrants. Overall, the quadrants display relatively distinct differential structures along the Shape and Body/Space dimensions. Both the Positive High and Positive Low quadrants exhibit elevated responses in selected Body/Space features, indicating that positive-emotion movements are more readily manifested through patterns of bodily expansion and spatial organization. In contrast, the Negative Low quadrant shows attenuated responses in certain Body/Space dimensions while displaying higher mean patterns along the Effort dimension. This observation suggests that sequence-level Effort means are not necessarily equivalent to psychological arousal; rather, they may be modulated by movement amplitude, rhythmic transitions, and shifts in the body’s center of gravity. Consequently, Effort-related dynamic information is better examined through subsequent window-level sensitivity analyses or investigations of emotion-intensity variation.

#### 4.2.2. Time-Window Aggregation Sensitivity Analysis

To examine the influence of temporal scale on LMA–RCM modeling, we perform a temporal-window aggregation sensitivity analysis. Rather than treating short windows as independent samples, each dance sequence is first partitioned into non-overlapping windows of varying lengths, and the mean LMA features are computed within each window. The means and standard deviations of all windows belonging to the same sequence are then aggregated into sequence-level statistical descriptors. Supervision remains at the full-sequence level, thereby avoiding label noise introduced by local windows.

Table 5 reports four-quadrant classification and valence–arousal mapping results for the sequence-level mean and different window-aggregation settings. The mapping is scale-dependent. The 240-frame aggregate gave the highest quadrant macro-F1 (0.618±0.125); the 30-frame aggregate gave the highest valence and arousal $R^{2}$ (0.403±0.193, 0.499±0.234). Longer windows suppress local noise and help coarse region labels; shorter windows keep accents that continuous coordinates use. A single sequence-level mean is therefore not a neutral summary. The preferred scale differs by task. Resolving that trade-off needs phrase boundaries or a sequential model, not a finer grid of the same global mean (Section 5).

#### 4.2.3. Ablation Study of LMA Components

To assess the contributions of the implemented LMA-derived feature groups to dance emotion modeling, we conduct ablation experiments under fixed classifier/regressor configurations and data partitions, varying only the input feature composition. Body and Space are represented by a combined feature group in these experiments and are not evaluated separately. Five experimental conditions are examined: Effort only, Shape only, Body/Space only, Effort+Shape, and Full LMA. Performance is evaluated on both RCM four-quadrant classification and continuous valence–arousal mapping.

As shown in Table 6, Full LMA achieves the best overall performance (macro-F1 0.565±0.070, valence $R^{2}$ 0.266±0.273, arousal $R^{2}$ 0.403±0.189). Among single-component variants, Body/Space only yields the strongest results (macro-F1 0.542±0.116), approaching the full feature set and retaining substantial explanatory power on the continuous mapping task. This indicates that joint relative relations under root-node reference, together with spatial organizational patterns, constitute the primary discriminative source within the current LMA representation.

**Figure 4:**
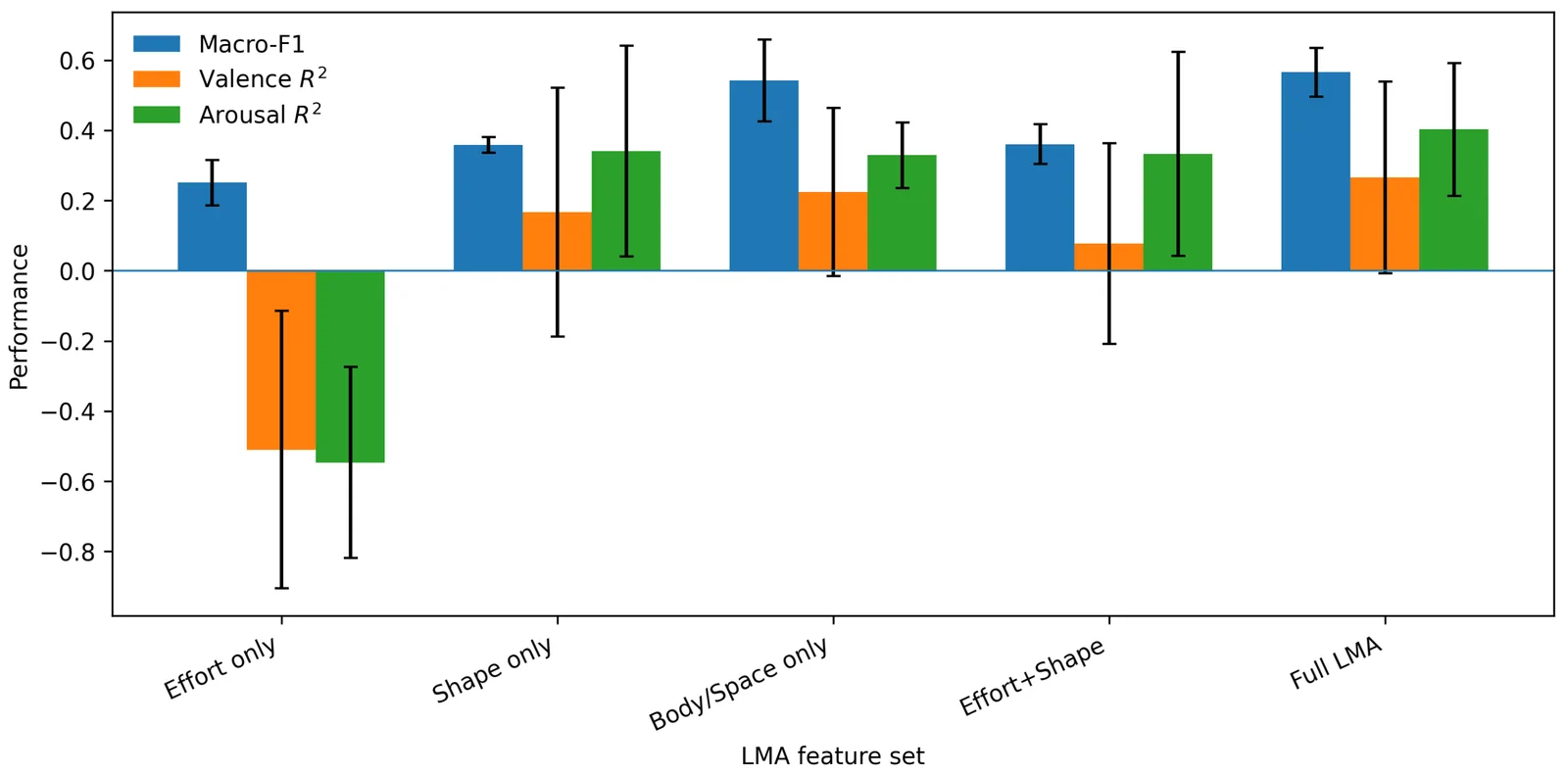
Ablation results of different LMA component combinations for RCM quadrant classification and valence–arousal mapping. Bars show the mean performance across cross-validation folds, and error bars indicate standard deviations.

As shown in Figure 4, Effort-only input was close to chance (macro-F1 0.251±0.065) and yielded negative valence and arousal $R^{2}$ (−0.510±0.396, −0.547±0.272). Under sequence-level averaging, the four Effort channels therefore add no usable coordinate information and can pull the fit below a constant predictor.

The Effort-only collapse should not be read as evidence that Laban Effort is irrelevant to dance affect. In this implementation, the four channels are weighted copies of one joint-acceleration statistic (Equation (5)), which are then averaged over several thousand frames. That operator removes attack, sustain, and recovery—the quantities Effort is meant to describe. The negative $R^{2}$ may reflect an interaction between a redundant descriptor and a pooling choice, not a test of the Effort factors as defined in LMA. The windowed aggregates in Table 5 point in the same direction: once local variation is restored, continuous mapping improves. A direct Effort-by-window ablation was not run; the interpretation is taken up in Section 5.

### 4.3. RCM-Based Emotion Classification and Prototype Mapping Performance

After completing the statistical analysis of LMA features, the emotion-modeling capability of the LMA-derived representations is evaluated at two levels: RCM four-quadrant classification and continuous valence–arousal prototype mapping. The former examines the ability of dance-movement features to discriminate the basic affective regions defined by valence and arousal, while the latter assesses their predictive performance on continuous RCM emotion coordinates. In contrast to fine-grained emotion-word classification, this section focuses on the capacity of LMA representations to recover the fundamental affective structure of the RCM space. First, the original fine-grained emotion labels are mapped onto the four quadrants—Positive High, Positive Low, Negative High, and Negative Low—according to the RCM model, and four-quadrant classification experiments are conducted.

Table 7 reports the results obtained by different classification algorithms under identical sequence-level LMA feature inputs. Given the class imbalance among the four quadrants, macro-F1 is adopted as the primary evaluation metric to reduce the bias of accuracy toward the majority class. The results show that SVM-RBF achieves the best overall performance, with an accuracy of 0.596±0.050 and a macro-F1 of 0.565±0.070. Random Forest attains the same accuracy but yields a lower macro-F1 and a larger standard deviation, indicating weaker stability across folds. MLP performs substantially worse, suggesting that, under the present small-sample sequence-level feature conditions, more complex nonlinear neural networks do not confer a performance advantage.

**Figure 5:**
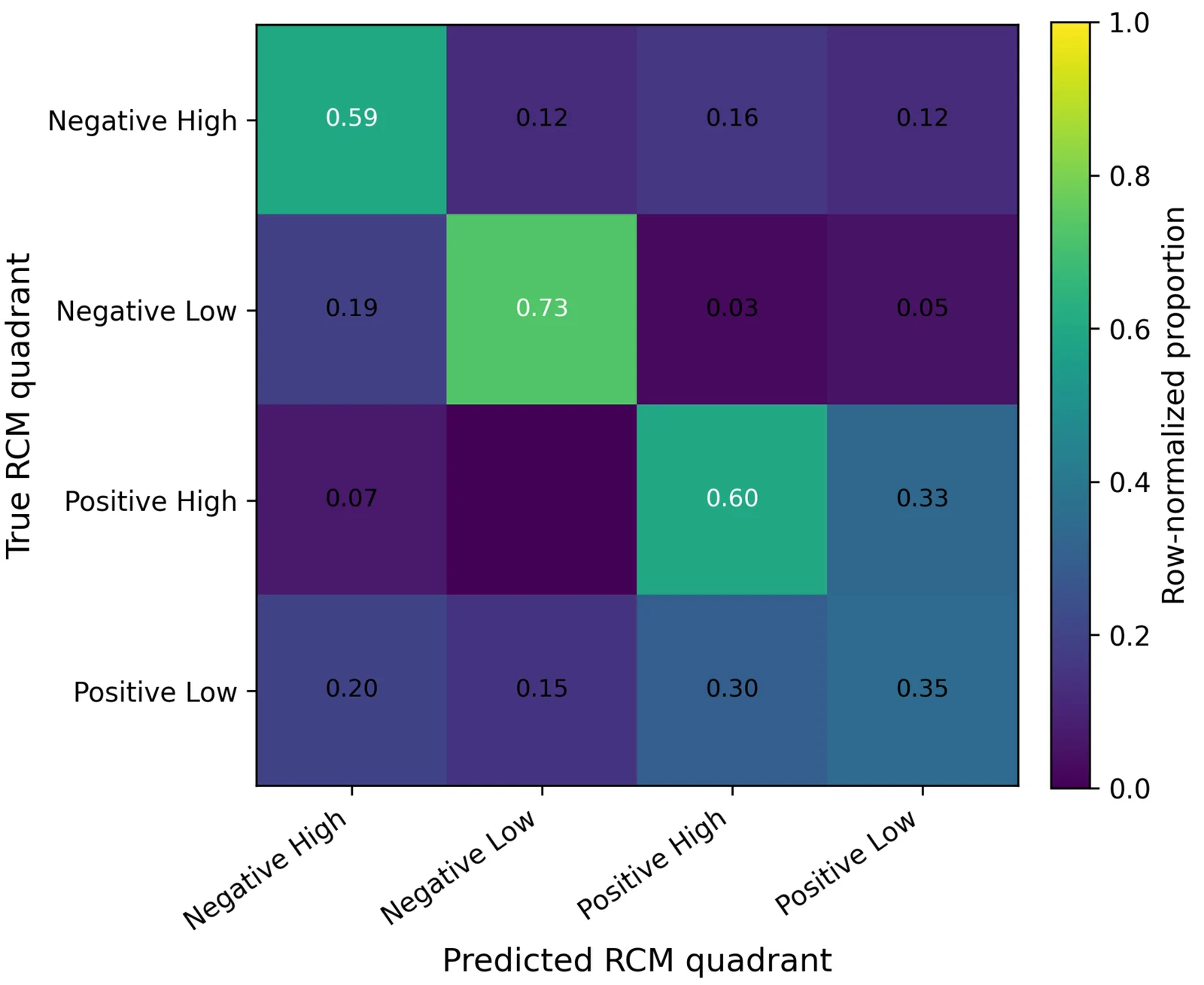
Row-normalized confusion matrix of the best-performing classifier for RCM quadrant prediction using sequence-level LMA features. Rows indicate true RCM quadrants, and columns indicate predicted RCM quadrants. Values represent the proportion of samples in each true quadrant assigned to each predicted quadrant.

Figure 5 presents the row-normalized confusion matrix of the SVM-RBF classifier. Diagonal accuracies for Negative Low, Positive High, and Negative High reach 0.73, 0.60, and 0.59, respectively, indicating that sequence-level LMA features can discriminate the primary affective regions. Positive Low, however, attains only 0.35 accuracy and is frequently misclassified as Positive High. Positive-valence movements share similar patterns in body shape and spatial organization; arousal differences within the same valence polarity therefore still require finer-grained temporal dynamic features for reliable separation.

We further evaluate the regression performance of sequence-level LMA features onto RCM valence–arousal prototype coordinates. Unlike four-quadrant classification, this task maps movements directly into the two-dimensional continuous affective space, thereby testing the capacity of LMA representations to support continuous emotion prototypes. Table 8 shows that Random Forest regression achieves the best overall result, with a mean $R^{2}$ of 0.334. The valence score is $R^{2}=0.266\pm 0.273$ and the arousal score is $R^{2}=0.403\pm 0.189$. Arousal substantially outperforms valence, indicating that LMA features are more sensitive to activation-related motion and only partially capture valence polarity. Compared with OLS and ridge, Random Forest yields a more favorable trade-off between error and explanatory power, particularly on valence, suggesting a nonlinear relationship between the features and affective coordinates. The large standard deviation of the valence $R^{2}$, however, reveals insufficient cross-fold stability; the present representation therefore recovers only partial valence information.

### 4.4. Analysis of the Circumplex Structure of Affective Space in RCM

To further verify whether dance movement features can support structured modeling of the continuous RCM emotion space, this study introduces enhanced temporal statistical LMA features together with circular target regression, building upon four-quadrant classification and direct VA regression. Rather than predicting valence and arousal directly in Cartesian coordinates, the circular mapping represents emotion prototypes as a combination of direction and intensity by separately estimating cosθ, sinθ, and intensity *r*, which are subsequently reconstructed into two-dimensional RCM coordinates. This formulation explicitly encodes the directional structure of the emotion circumplex and thereby alleviates the center-contraction problem frequently observed in direct VA regression. Concurrently, the input representation is expanded from sequence-level means to a richer set of temporal statistics—including standard deviation, quantiles, variation amplitude, energy, and temporal trends—so as to more effectively capture dynamic differences among movement sequences.

Table 9 shows that ExtraTrees attains the best overall performance when trained on the full set of enhanced features: valence $R^{2}=0.437\pm 0.086$, arousal $R^{2}=0.304\pm 0.361$, angle MAE of 36.765 ± 6.414°, and quadrant macro-F1 of 0.670 ± 0.094. Relative to direct VA regression and four-quadrant classification, the enhanced circular mapping yields a clear improvement in valence prediction and, after remapping onto quadrants, produces a higher macro-F1 score, demonstrating that the circular target representation helps recover the directional organization of emotion within RCM space.

**Figure 6:**
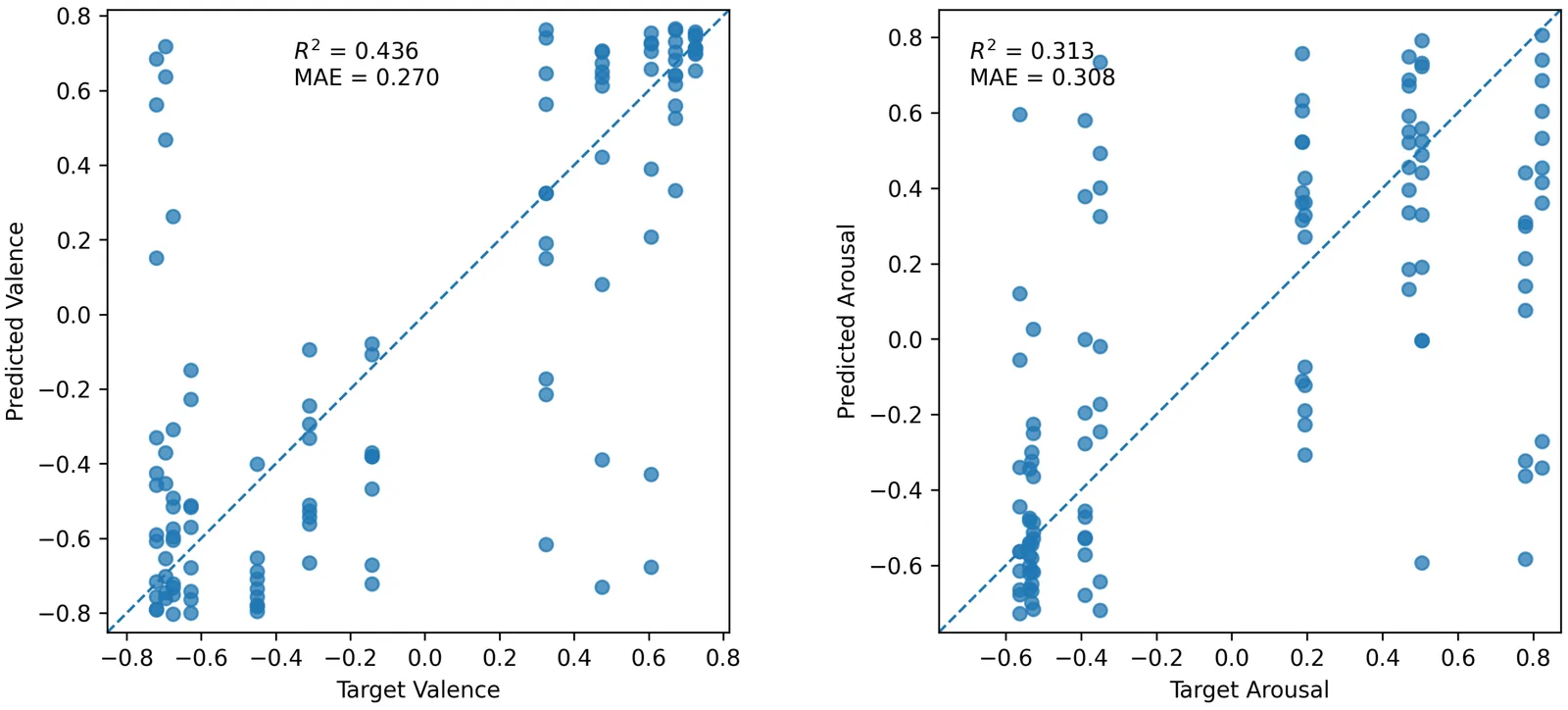
Predicted versus target RCM coordinates obtained from the enhanced circular mapping model. The dashed line indicates the ideal prediction, and each point represents one dance sequence.

Figure 6 presents the prediction performance of the enhanced circular mapping model on continuous VA coordinates. The valence dimension exhibits a clear positive correlation, with a pooled $R^{2}$ of 0.436 and an MAE of 0.270, indicating that the enhanced LMA temporal statistical features effectively discriminate positive and negative affective directions. Performance on the arousal dimension is comparatively weaker, yielding a pooled $R^{2}=0.313$ and an MAE of 0.308. This suggests that the model retains a moderate continuous predictive capability for high and low arousal levels, although its stability remains lower than that of valence. These results align with the cross-validation outcomes reported in Table 9, confirming that the enhanced circular mapping improves the overall predictive performance on continuous emotion coordinates, while differences in modeling difficulty across the two dimensions persist.

**Figure 7:**
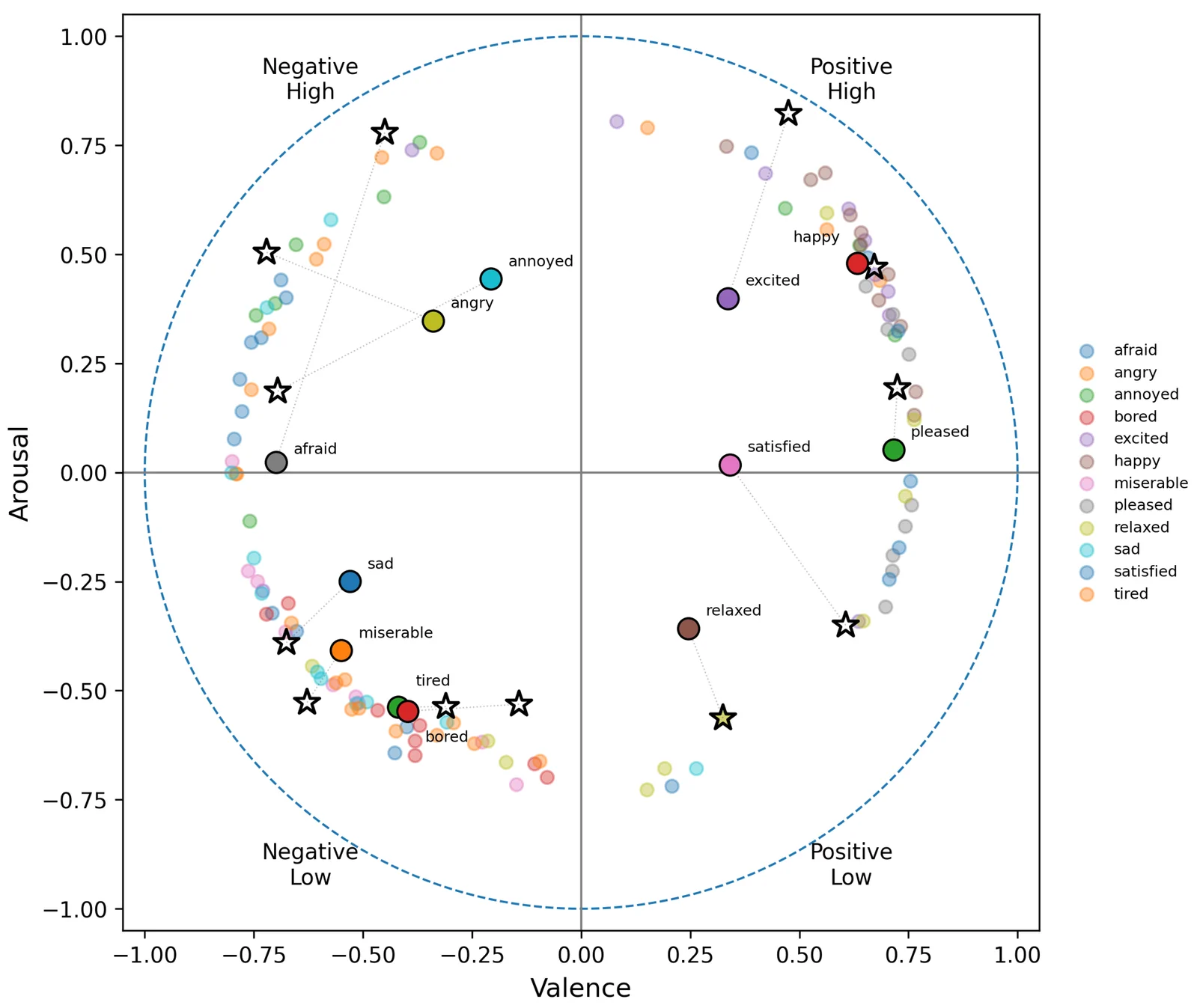
Predicted distribution of dance sequences in the RCM valence–arousal space using enhanced temporal LMA features and circular target regression. Star markers indicate predefined RCM emotion prototypes, filled circles indicate predicted emotion centroids, and dotted lines show deviations between target prototypes and predicted centroids.

From a spatial-structure perspective, Figure 7 shows that the predicted samples obtained after enhanced circular mapping no longer concentrate markedly at the center of the RCM space. Instead, they form a clearer structured distribution across the four quadrants. Positive high-arousal emotions are predominantly located in the upper-right region, and negative high-arousal emotions in the upper-left region; negative low-arousal emotions are concentrated in the lower-left region, and positive low-arousal emotions are restored to the lower-right region. This distribution indicates that the combination of enhanced temporal statistical features and circular target regression effectively mitigates the center-contraction effect and produces predictions that better conform to the directional organization of the RCM affective space.

Further examination at the emotion-category level reveals that the enhanced circular mapping does not recover all emotion prototypes with equal fidelity. As shown in Table 10, the circular-mean angular deviations for happy, relaxed, miserable, and sad remain below 3°, indicating that the mean predicted directions of these categories are close to their assigned prototype angles. Pleased, excited, angry, and tired also exhibit relatively small angular deviations. In contrast, afraid, annoyed, and satisfied produce larger angular errors of 59.2°, 50.3°, and 30.4°, respectively. These results show that the model successfully recovers the principal directional structure of the RCM space, while predictions for boundary or more semantically complex emotion categories remain less stable.

The mapping therefore recovers the coarse four-quadrant layout more reliably than the 12 listed prototypes. Afraid is recovered near negative-neutral rather than negative-high (179.2° vs. 120°); annoyed shifts toward the afraid/angry sector (114.7° vs. 165°); satisfied collapses toward the positive-neutral axis (0.4° vs. 330°). Two properties of the feature set help explain the pattern. First, the implemented Effort channels are four weighted functions of the same joint-acceleration statistic; sequence-level pooling of those channels is a poor substitute for Time, Weight, Flow, and Space as Laban factors, and the ablation already shows Effort-only performance at chance. Boundary labels in this vocabulary differ less by gross expansion than by attack, weight, and phrase shape—quantities the 31 descriptors resolve only weakly. Second, Body/Space dominates the ablation, so categories that share root-referenced pose and bounding volume (positive-low vs. positive-high; annoyed vs. angry/afraid) remain close in input space after temporal aggregation. The questionnaire agreement rates move in the same direction (afraid 0.48, annoyed 0.44, satisfied 0.54), which is consistent with label ambiguity in the recordings themselves, not only with regressor error.

The same assigned prototypes can be connected by the polar interpolant of §3.1.2. The question here is geometric: whether shortest-angle paths between listed (θ,r) targets remain continuous on the circumplex and avoid the origin. This is not a test of generated movement and does not use predicted centroids as endpoints. Endpoints are the predefined prototypes in Table 1; prediction error is discussed separately in Table 10.

**Figure 8:**
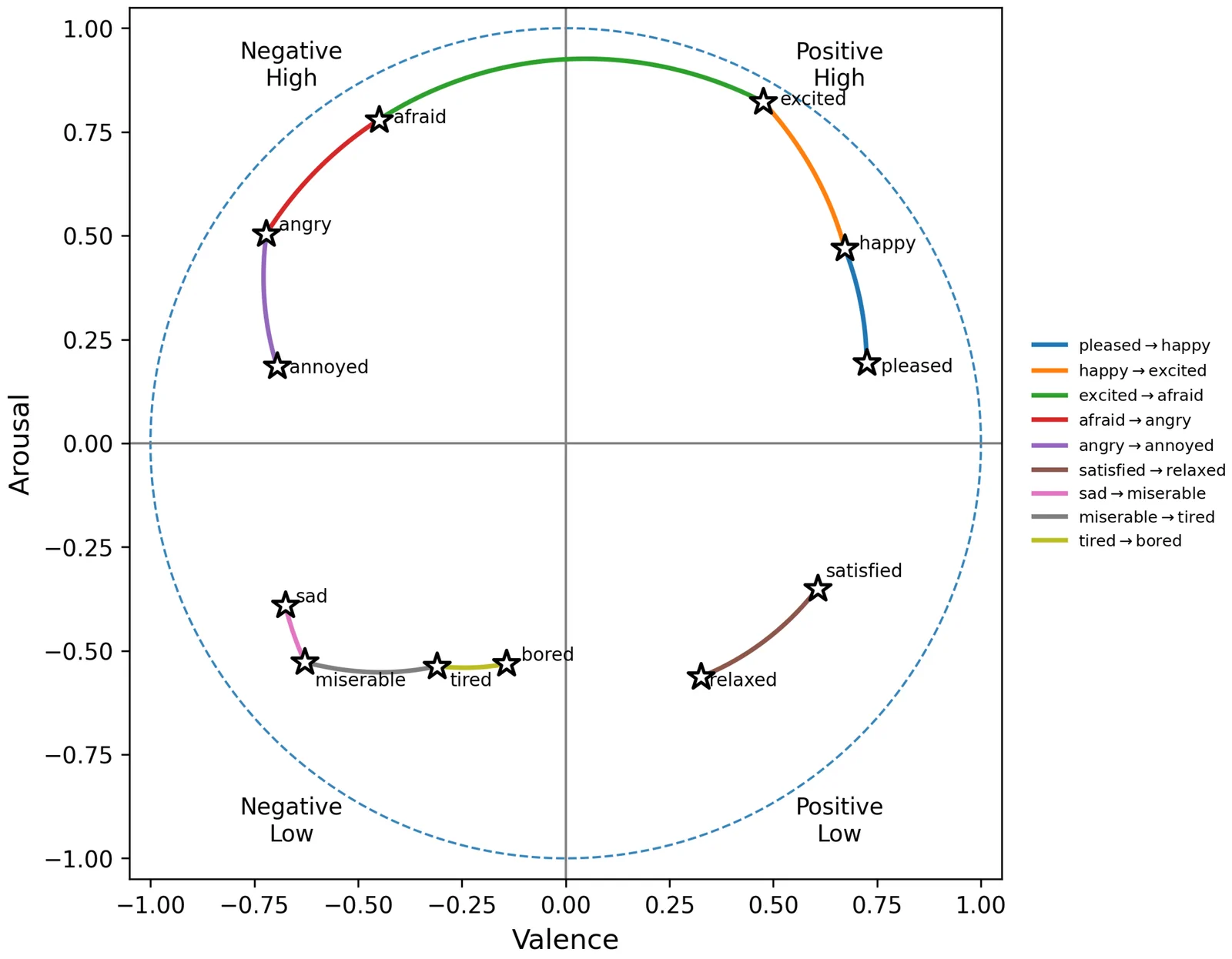
Circular transition trajectories between representative emotion prototypes in the RCM space. Each trajectory was generated by interpolating affective angle and intensity along the shortest angular path between source and target prototypes. Star markers indicate predefined RCM emotion prototypes, and the dashed circle indicates the reference affective ring.

Figure 8 shows polar interpolation paths between the predefined prototypes in Table 1. The within-quadrant paths and the cross-quadrant excited → afraid path remain outside the origin. For the nine paths sampled at K=80, Table 11 reports max jump from 0.1266° (sad → miserable) to 0.7595° (excited → afraid) and smoothness from 0.000005 to 0.000163 for the same respective paths.

For the nine prototype paths in Table 11, max jump ranges from 0.1266° for sad → miserable to 0.7595° for excited → afraid. Smoothness ranges from 0.000005 to 0.000163 for the same respective paths. Under uniform shortest-angle sampling, max jump equals |Δθ|/(K−1); smoothness is the mean Euclidean norm of the second differences of the sampled (V,A) coordinates. Both metrics characterize the predefined prototype paths at K=80.

### 4.5. Results of Subjective Perceptual Validation

Subjective perception validation results indicated that participants’ ratings of valence, arousal, and clarity of emotional expression for the dance segments exhibited acceptable consistency. Cronbach’s α values for valence, arousal, and clarity were 0.84, 0.79, and 0.76, respectively, indicating that the subjective evaluations were not random responses but reflected relatively stable perceptual trends while preserving individual differences. Repeated-measures analysis of variance further revealed significant differences among the target emotion categories on all three subjective ratings. Because Mauchly’s test of sphericity indicated that the sphericity assumption was violated, Greenhouse–Geisser-corrected statistics are reported. Post hoc comparisons employed Bonferroni or Benjamini–Hochberg FDR corrections to control the false-positive risk arising from multiple comparisons.

**Figure 9:**
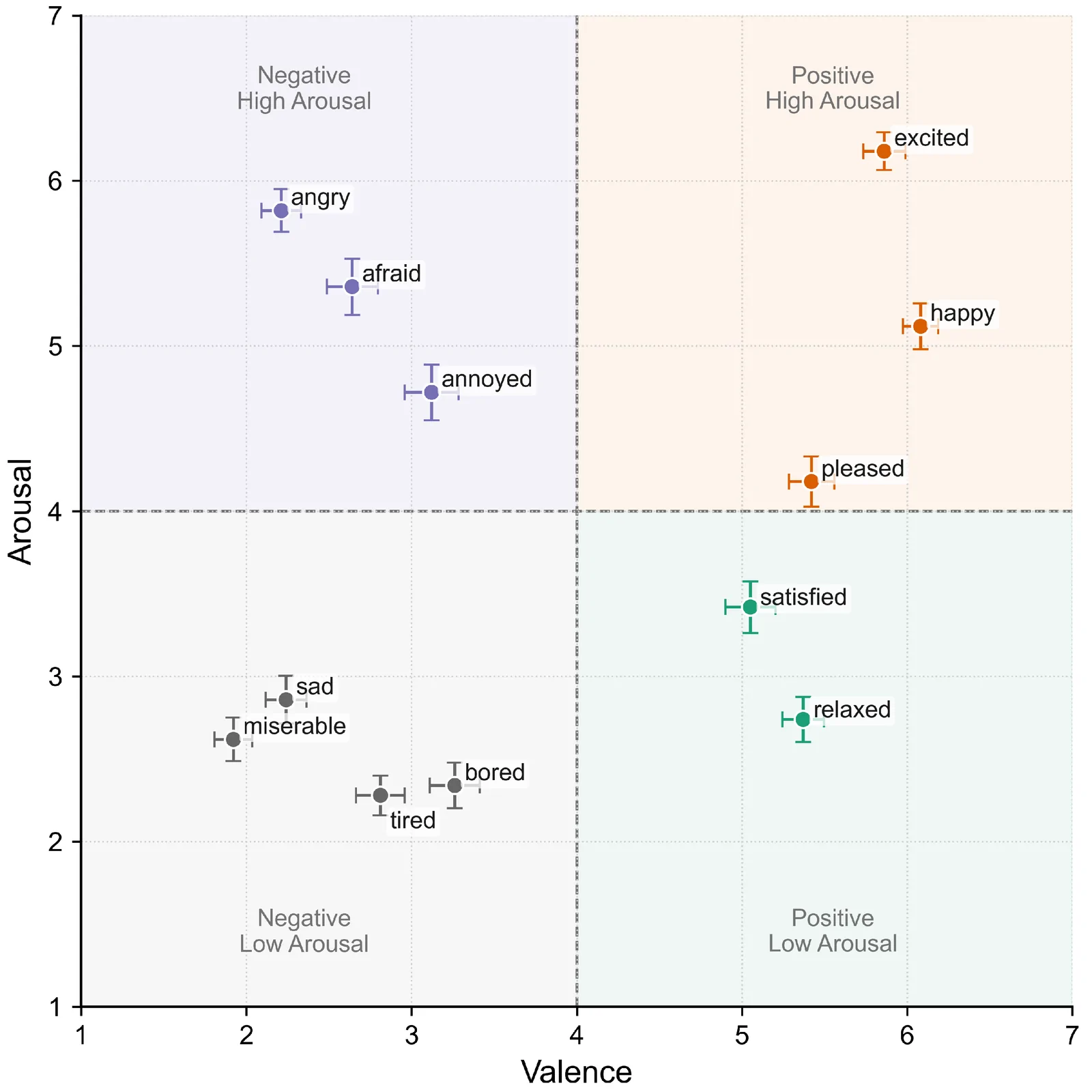
Subjective valence–arousal distribution of the 12 emotion categories. Each point denotes the mean participant rating for one emotion category, with horizontal and vertical error bars representing the standard errors of the mean for valence and arousal, respectively. The dashed lines indicate the neutral midpoint of the seven-point Likert scales. Shaded regions indicate the four RCM quadrants.

From the distribution in valence–arousal space, the subjective ratings were broadly consistent with the predefined RCM quadrant structure. As shown in Figure 9, happy and excited occupied the positive high-arousal region, and relaxed and satisfied the positive low-arousal region, while angry, afraid, and annoyed were predominantly located in the negative high-arousal region; sad, miserable, tired, and bored clustered in the negative low-arousal region. Detailed descriptive statistics are reported in Table 12. Several categories nevertheless depart from idealized quadrant separation: the mean arousal of pleased lies near the neutral midpoint, annoyed is closer to the RCM center than angry or afraid, and the mean valence of bored approaches the midpoint. Category-selection agreement is lower for afraid, annoyed, satisfied, and bored than for excited, happy, angry, relaxed, and miserable.

Further correlation analysis reveals moderate agreement between the model-predicted RCM coordinates and the subjective perception scores. Predicted valence is significantly correlated with mean subjective valence (Pearson’s r=0.62, p<0.001); predicted arousal is significantly correlated with mean subjective arousal (r=0.48, p=0.006); and predicted angle is significantly correlated with the subjectively transformed angle (r=0.55, p=0.002). In addition, the mean clarity score of each clip is significantly negatively correlated with the model’s angular error (r=−0.41, p=0.018), indicating that clips with clearer emotional expression tend to have predicted directions closer to the predefined RCM prototypes. Overall, the ratings are consistent with the assigned four-quadrant layout and mark afraid, annoyed, satisfied, and bored as less stable labels. The protocol used short 2D RGB excerpts of the original dancers. The result therefore concerns segment-level label appropriateness under full visual appearance. It does not isolate kinematics, and it does not speak to multi-minute improvisation, synthesized skeletons, or immersive viewing. The 12–16.5 s clip duration may give greater weight to salient local gestures than to gradual affective development; this possibility was not tested because clip duration was not varied.

## 5. Discussion

This study examines the use of the continuous RCM affective space for organizing predefined emotion targets in recorded modern dance, while LMA-informed kinematic descriptors provide inputs for prediction. The valence–arousal representation expresses the assigned angular direction and radius of each category and permits evaluation of angular error and quadrant membership. The enhanced circular model achieved the reported angular and quadrant scores on its 12-category dataset, but its comparison with direct VA regression does not isolate the effect of circular targets because the two experiments used different prototype tables, samples, and input features.

The feature-group analysis indicates that the implemented kinematic descriptors differ in predictive utility for this dataset. In the reported ablation, the combined Body/Space group was the strongest single group, Shape yielded intermediate results, and the four acceleration-based Effort proxies performed less well. The four Effort channels are weighted copies of one acceleration statistic, and sequence averaging removes the timing of local changes. Their weak standalone performance may reflect both descriptor redundancy and temporal pooling. Because the window comparison used the full feature set, an Effort-specific benefit of shorter windows remains untested; phrase-aligned or sequential features could help examine this possibility. The prominence of Body/Space cues may partly reflect the acted recordings, in which prescribed emotions can encourage recognizable postural patterns. In less constrained dance, similar affective states may be conveyed through subtler, dancer-specific timing and movement phrasing. Given the small number of sequences per category and the absence of dancer- or choreography-disjoint evaluation, whether this feature pattern extends to unseen performers and movement styles remains uncertain. Four-quadrant classification together with direct VA mapping shows that the LMA representation possesses moderate discriminative and predictive capacity; these results do not establish that Cartesian regression generally fails to represent circular affective structure. The enhanced circular model predicts cosθ, sinθ, and *r* for assigned category targets, so its output encodes periodic direction; it does not recover a frame-by-frame emotional trajectory from a dance sequence.

At the category level, circular-mean angular deviations were small for happy, relaxed, miserable, and sad, but reached 59.2°, 50.3°, and 30.4° for afraid, annoyed, and satisfied, respectively. In the separate questionnaire on videos of the original dancers, observer ratings broadly followed the predefined quadrants, while category agreement was lower for afraid, annoyed, and satisfied. This descriptive correspondence does not establish the cause of the model errors or how interpolated movement would be perceived. The prototype interpolation produced continuous numerical paths in affective-coordinate space; the jump and second-difference measures in Table 11 describe these paths at the fixed sampling resolution of K=80 and do not establish the perceptual smoothness of skeletal motion.

## 6. Conclusions

This study maps LMA-informed kinematic descriptors from recorded modern dance onto assigned circular targets in Russell’s valence–arousal space. On the 12-category set, ExtraTrees yielded an angular MAE of 36.765 ± 6.414° and a quadrant macro-F1 of 0.670 ± 0.094. Body/Space features were the strongest single feature group, whereas the sequence-level Effort features performed poorly. This result may reflect both temporal aggregation and the fact that the four implemented Effort channels share one acceleration statistic. The reported scores describe the recovery of assigned category targets.

Interpolation traces numerical paths between predefined prototypes. The questionnaire concerns 2D RGB videos of the original dancers and does not separate kinematics from appearance or scene context. The present study did not evaluate sequential affect prediction, generated poses, or real-time immersive playback. Future work should assess affective transitions in generated skeletal motion, test their perception with controlled avatar stimuli, and examine sequential modeling and real-time performance.

## Figures and Tables

**Table 1:** Emotion prototypes and movement characteristics in the RCM space.

Emotion	θ	*r*	Quadrant	Movement Characteristics
Pleased	15°	0.75	PH	Gentle expansion, soft spine extension, and slow fluid movement.
Happy	35°	0.82	PH	Upward torso extension, elastic steps, and rhythmic arm swings.
Excited	60°	0.95	PH	Rapid arm lifting, bouncing steps, and energetic large-amplitude movement.
Afraid	120°	0.90	NH	Inward contraction, forward lean, protective arms, and sudden withdrawal.
Angry	145°	0.88	NH	Powerful direct movement, forward torso, forceful arm swings, and heavy steps.
Annoyed	165°	0.72	NH	Slight rigidity, abrupt movement, shrugged shoulders, and rejecting gestures.
Sad	210°	0.78	NL	Forward-bent spine, slow contraction, lowered limbs, and closed posture.
Miserable	220°	0.82	NL	Strong inward curling, heavy forward lean, and slow weak movement.
Tired	240°	0.62	NL	Slow heavy movement, lowered shoulders, forward torso, and dragging limbs.
Bored	255°	0.55	NL	Sinking posture, repetitive movement, limited extension, and low energy.
Relaxed	300°	0.65	PL	Natural expansion, slow flowing movement, and freely extended limbs.
Satisfied	330°	0.70	PL	Gentle torso expansion, steady movement, and comfortable stable posture.

Note: PH = positive high arousal; PL = positive low arousal; NH = negative high arousal; NL = negative low arousal. The movement descriptions illustrate the intended category meanings; they were not used as model inputs or independently validated movement annotations.

**Table 2:** Demographic characteristics of participants in the subjective perceptual validation questionnaire.

Variable	Category	*N* (%)
Age group	18–24 years	13 (36.1%)
	25–30 years	12 (33.3%)
	31–39 years	10 (27.8%)
	≥40 years	1 (2.8%)
Gender	Female	17 (47.2%)
	Male	17 (47.2%)
	Prefer not to say	2 (5.6%)
Dance experience	None	13 (36.1%)
	Basic	13 (36.1%)
	Intermediate	7 (19.4%)
	Advanced	3 (8.3%)

**Table 3:** Statistical summary of the Custom Acted Dance Motion Capture Dataset.

Emotion	Samples	Mean Frames	Std Frames	Mean Duration (s)	Min Frames	Max Frames
active	1	5137.00	0.00	171.23	5137	5137
afraid	9	7986.22	4614.07	266.21	2345	13,385
angry	12	6869.08	4250.67	228.97	2300	13,166
annoyed	9	6582.11	4528.97	219.40	2221	12,415
bored	8	7434.38	4237.78	247.81	2774	13,199
curious	1	10,613.00	0.00	353.77	10613	10,613
excited	10	7030.60	4680.08	234.35	601	12,697
happy	11	7400.27	4174.18	246.68	2582	12,723
miserable	9	7076.78	4106.57	235.89	3038	12,576
mix	5	12,641.20	6323.13	421.37	76	16,384

**Table 4:** Top LMA features showing significant differences across the four RCM affective quadrants after FDR correction.

Feature	Component	*H*	FDR *p*	$\epsilon^{2}$
lma_dim_4	Shape	45.730	<0.001	0.372
lma_dim_12	Body/Space	39.723	<0.001	0.319
lma_dim_15	Body/Space	39.723	<0.001	0.319
lma_dim_6	Shape	36.567	<0.001	0.292
lma_dim_24	Body/Space	34.654	<0.001	0.275
lma_dim_26	Body/Space	34.654	<0.001	0.275
lma_dim_27	Body/Space	34.654	<0.001	0.275
lma_dim_29	Body/Space	34.654	<0.001	0.275
lma_dim_30	Body/Space	27.769	<0.001	0.215
lma_dim_18	Body/Space	27.301	<0.001	0.211

**Table 5:** Sensitivity analysis of temporal window aggregation for RCM quadrant classification and valence–arousal mapping.

Condition	Sequences	Mean Windows	Feature Dim.	Macro-F1	Valence $R^{2}$	Arousal $R^{2}$
Sequence-level mean	131	1.0	31	0.565±0.070	0.266±0.273	0.403±0.189
30-frame aggregation	131	244.6	62	0.567±0.157	0.403±0.193	0.499±0.234
60-frame aggregation	131	122.3	62	0.527±0.107	0.391±0.164	0.437±0.237
120-frame aggregation	131	61.2	62	0.606±0.129	0.377±0.211	0.383±0.216
240-frame aggregation	130	30.8	62	0.618±0.125	0.348±0.256	0.447±0.179

**Table 6:** Ablation results of different LMA component combinations for RCM quadrant classification and valence–arousal mapping.

Feature Set	Dim.	Macro-F1	Valence $R^{2}$	Arousal $R^{2}$
Full LMA	31	0.565 ± 0.070	0.266 ± 0.273	0.403 ± 0.189
Body/Space	23	0.542 ± 0.116	0.224 ± 0.240	0.329 ± 0.093
Effort+Shape	8	0.361 ± 0.056	0.077 ± 0.286	0.333 ± 0.291
Shape	4	0.359 ± 0.022	0.166 ± 0.355	0.341 ± 0.300
Effort	4	0.251 ± 0.065	−0.510 ± 0.396	−0.547 ± 0.272

**Table 7:** Classification performance of different algorithms using sequence-level LMA features for RCM quadrant prediction.

Model	Accuracy	Precision	Recall	Macro-F1
SVM-RBF	0.596 ± 0.050	0.589 ± 0.074	0.567 ± 0.069	0.565 ± 0.070
Random Forest	0.596 ± 0.133	0.559 ± 0.168	0.553 ± 0.134	0.531 ± 0.152
Logistic Regression	0.521 ± 0.110	0.528 ± 0.107	0.499 ± 0.122	0.492 ± 0.118
MLP	0.404 ± 0.040	0.287 ± 0.102	0.346 ± 0.055	0.280 ± 0.087

**Table 8:** Regression performance of different models for mapping sequence-level LMA features to RCM valence–arousal coordinates.

Model	Valence MAE	Valence RMSE	Valence $R^{2}$	Arousal MAE	Arousal RMSE	Arousal $R^{2}$
Random Forest Regression	0.381±0.055	0.483±0.086	0.266±0.273	0.366±0.048	0.488±0.094	0.403±0.189
Ridge Regression	0.425±0.054	0.522±0.081	0.147±0.276	0.379±0.049	0.480±0.064	0.408±0.204
OLS	0.443±0.057	0.548±0.081	0.058±0.316	0.386±0.057	0.487±0.071	0.386±0.236

**Table 9:** Performance of the top enhanced circular mapping models for predicting RCM affective coordinates from temporal LMA features.

Model	*K*	Valence $R^{2}$	Arousal $R^{2}$	Angle MAE (°)	Intensity MAE	Quadrant Macro-F1
ExtraTrees	all	0.437±0.086	0.304±0.361	36.765±6.414	0.081±0.011	0.670±0.094
ExtraTrees	150	0.361±0.137	0.256±0.346	39.378±6.636	0.082±0.010	0.684±0.062
ExtraTrees	100	0.363±0.160	0.273±0.373	39.357±8.923	0.081±0.012	0.667±0.100
Random Forest	100	0.423±0.165	0.231±0.303	37.719±7.636	0.080±0.010	0.650±0.083
Random Forest	all	0.479±0.156	0.132±0.433	38.567±6.590	0.083±0.009	0.644±0.099

**Table 10:** Category-level circular-mean angular deviations and Cartesian centroid errors under the enhanced circular mapping model.

Emotion	*N*	Target Angle (°)	Pred. Angle (°)	Angle Error (°)	Centroid Error
pleased	9	15.0	3.7	11.3	0.142
happy	11	35.0	36.7	1.7	0.040
excited	10	60.0	48.8	11.2	0.446
relaxed	11	300.0	302.0	2.0	0.220
satisfied	9	330.0	0.4	30.4	0.453
afraid	9	120.0	179.2	59.2	0.796
angry	12	145.0	135.4	9.6	0.413
annoyed	9	165.0	114.7	50.3	0.552
sad	11	210.0	207.3	2.7	0.202
miserable	9	220.0	217.7	2.3	0.142
tired	9	240.0	232.8	7.2	0.109
bored	8	255.0	234.8	20.2	0.255

**Table 11:** Quantitative metrics of circular transition trajectories between representative emotion prototypes.

Transition	Start	End	Max Jump	Smoothness
	(°)	(°)	(°)	
pleased → happy	15.0	35.0	0.2532	0.000017
happy → excited	35.0	60.0	0.3165	0.000033
excited → afraid	60.0	120.0	0.7595	0.000163
afraid → angry	120.0	145.0	0.3165	0.000027
angry → annoyed	145.0	165.0	0.2532	0.000024
satisfied → relaxed	330.0	300.0	0.3797	0.000031
sad → miserable	210.0	220.0	0.1266	0.000005
miserable → tired	220.0	240.0	0.2532	0.000026
tired → bored	240.0	255.0	0.1899	0.000009

**Table 12:** Subjective perceptual validation results by emotion category.

Emotion	Valence	Arousal	Clarity	Agreement
pleased	5.42 ± 0.82	4.18 ± 0.91	4.96 ± 0.88	0.61
happy	6.08 ± 0.64	5.12 ± 0.83	5.74 ± 0.71	0.76
excited	5.86 ± 0.77	6.18 ± 0.69	5.68 ± 0.73	0.79
relaxed	5.37 ± 0.76	2.74 ± 0.82	5.31 ± 0.80	0.72
satisfied	5.05 ± 0.91	3.42 ± 0.94	4.62 ± 0.93	0.54
angry	2.21 ± 0.72	5.82 ± 0.78	5.58 ± 0.76	0.73
afraid	2.64 ± 0.93	5.36 ± 1.02	4.51 ± 1.05	0.48
annoyed	3.12 ± 0.98	4.72 ± 1.01	4.38 ± 0.96	0.44
sad	2.24 ± 0.74	2.86 ± 0.87	5.11 ± 0.82	0.68
miserable	1.92 ± 0.69	2.62 ± 0.79	5.27 ± 0.76	0.71
tired	2.81 ± 0.88	2.28 ± 0.72	4.89 ± 0.86	0.63
bored	3.26 ± 0.91	2.34 ± 0.83	4.57 ± 0.91	0.52

## Data Availability

The original contributions presented in this study are included in the article. Further inquiries can be directed to the corresponding author.
